# The Gut-Brain Axis and Its Role in Depression

**DOI:** 10.7759/cureus.10280

**Published:** 2020-09-06

**Authors:** Saruja Nanthakumaran, Saijanakan Sridharan, Manoj R Somagutta, Ashley A Arnold, Vanessa May, Sukrut Pagad, Bilal Haider Malik

**Affiliations:** 1 Department of Research, California Institute of Behavioral Neurosciences & Psychology, Fairfield, USA; 2 Surgery, California Institute of Behavioral Neurosciences & Psychology, Fairfield, USA; 3 Internal Medicine, California Institute of Behavioral Neurosciences & Psychology, Fairfield, USA

**Keywords:** gut-brain connection, depression, gut microbiomes, serotonin

## Abstract

The gut microbiota in humans communicates to the central nervous system through the gut-brain axis, and this communication functions in a bidirectional manner. The backbone of this axis is via the vagus nerve allowing the communication. Research on the functionality of the gut-brain axis is present; however, analysis of the diversity and stratification of the gut microbiota is in its infancy. Through the exploration of various studies focusing on the role of the gut microbiota and its effects on the efficacy of selective serotonin receptor inhibitors (SSRIs) in depression management, many promising alterations in constructive changes have emerged. It has become evident that a set of quantifiable microbial markers have been identified as consistent in the stools of depressive subjects that can be further used to determine the severity of disease progression - the presence of certain bacterial species being a common thread amongst the therapeutic bacteria for depression management. The vagus nerve's role in the gut-brain axis, which is vital to carry out any constructive alterations in the gut microbiota, has been strengthened through evidence of SSRIs depending on the vagus to execute therapeutic effects. This review will focus on the interaction between the diversity of the gut microbiota and investigate its link with depression.

## Introduction and background

Depression affects 21% of the global population yet is highly undiagnosed and left untreated for various reasons. Depression presents with a persistent low mood and is a common psychiatric disorder that faces the obstacles of social stigma, lack of effective therapies, and inadequate mental health resources [1]. The current first-line pharmacological treatment for major depressive disorders (MDD) is SSRIs [2]. The function of serotonin (5-hydroxytryptamine, 5-HT) would be considered pleiotropic as its influences can be seen in gastrointestinal, neurological/psychiatric, immune, and liver diseases [3,4]. There are several functions of 5-HT in the gut lumen, such as peristaltic reflexes contributing to gastrointestinal functioning. The largest population of 5-HT in the body is stored in the enterochromaffin (EC) cells of the gut epithelium. The release of 5-HT from the EC cells into the lumen is stimulated mechanically, allowing peristaltic reflexes; acids, bases, and nutrients, such as short-chain fatty acids and glucose, have also been able to create such a response [5].

The 400 to 1000 bacterial species harboring in the human gut also extend their role in the central nervous system. The focus on the gut microbiota's symbiotic relationship with its host has been gaining more attention [4]. The concept of the gut-brain axis has been a growing area of discussion. The exploration of gut microbiota having benefits towards behavior and brain function can yield advances in depression treatment [6,7,8]. Already in current practice, the treatment of anxiolytic and anti-depressive-like behavior has been mediated with* Lactobacillus rhamnosus JB-1 *oral therapy through the gut-brain axis [6]. The defined mechanism of SSRIs vastly highlights the role they play in the brain of preventing presynaptic neuronal reuptake of serotonin; however, the argument is made that the benefits of SSRIs are due to their role in the gut over that of the brain [6].

Further evaluation of how the gut microbiota affects the interactions of antidepressants with the gut-brain axis would allow further treatment and prevention opportunities for MDD patients. Gut microbiota has emerged as the new epicenter of interest of therapy for MDDs [9]. By analyzing the alterations of gut microbiota and its influences on the efficacy of SSRIs on the gut-brain axis, a better understanding of therapeutics of depressive and MDDs can be achieved.

## Review

Depressive flora

Gut microbiota, being stratified into healthy flora, has provided a platform to distinguish pathology from physiology in numerous incidents. Further differentiation of the pathological vegetation has allowed the concept of stool signatures. Many studies have shown that there is a difference in stool composition due to gut microbiota changes when comparing healthy subjects against depressive patients. Liu et al. conducted an experiment where they transplanted fecal microbiota from patients with depression and healthy individuals to germ-free rats. They found that the rats that received fecal microbiota from depressed patients displayed depression-like behaviour [7]. The induction of depression through fecal transplant highlights how manipulating gut microbiota can have an effect on brain chemistry. Fond et al. explores this manipulative concept by recommending fecal microbiota transplantation providing a new healthy gut microbiota as a treatment option for major depression and schizophrenia patients. Fond et al. argues microbiota-orientated treatments to be a promising approach to treatment in depression and schizophrenia and also states that the risks from fecal microbiota transplant procedure have been reduced through capsule administration [10]. Changes in stool composition are also evident in antidepressant treatments. In the study by Bharwani et al. stool samples were collected before treatment, then at three and six months after indicated changes from the antidepressive administrations [11]. Stool composition changes from antidepressant treatments suggest a correction to the gut flora of the depressive subjects, supporting the phenotyping of depressive flora. Zheng et al. further compare and contrast the difference in gut microbiota and conclude differences between the gut microbiota of MDD versus bipolar disease versus that of healthy controls. The gut microbiota of MDD subjects was characterized by enriched *Enterobacteriaceae *and *Alistipes *and decreased* Faecalibacterium*, *Coprococcus*, and *Dialister *compared to healthy controls who were matched for age, gender, and BMI; this study had two sets of controls where one was matched for demographic variables mentioned and the other was not to provide an assessment to real-world conditions. They also found that only *Pseudomonadaceae *levels differed in higher concentrations compared to healthy controls to bipolar subjects. A signature of 26 operational taxonomic units (OTUs) were determined to differentiate MDD from both bipolar subjects and healthy controls. These 26 markers can reflect the severity as four of the 26 microbial OTUs of the majority belonging to the *Lachnospiraceae *family were significantly associated with the Hamilton Depression Rating Scale (HAMD) in MDD or bipolar patients [12]. Broadening research to consider diet and pharmacotherapy can further assist in the understanding for an optimal environment for healthy gut microbiota. The current approaches identifying psychiatric diagnoses rely heavily on clinical interview and symptomatology. Further exploration of gut microbial signatures specific to mental conditions such as MDD can provide a quantifiable diagnostic test for an overall better cumulative standard towards providing a concrete diagnosis.

*Lactobacillus *an asset to the gut

Certain bacteria are singled out as part of the constructive measures towards decreased depressive presentations in terms of the metabolic pathways or the inhibitory effects on inflammatory markers. Many studies identify the addition of *Lactobacillus *to the gut microbiota to have beneficial effects on MDD patients [13]. Capuco et al. conducted a double-blinded study comparing the use of an SSRI and a probiotic containing *Lactobacillus Plantarum 299v* against a placebo. The results showed that the treatment group had decreased kynurenine concentrations, which is neurotoxic and neurodegenerative. Cognitive function also had improvements from the baseline in areas of attention, perceptivity, and verbal learning tasks. Tryptophan can engage in two metabolic pathways either towards serotonin or kynurenine. Tryptophan is shunted towards serotonin production via probiotics resulting in increased serotonin reducing depressive symptoms parallel to the mechanism of SSRIs [14]. Also, Han et al. makes it evident that the inhibition of nuclear factor kappa B (NF-κB) activation and tumor necrosis factor alpha (TNFα), which play a role in the pathogenesis of depression in anxiety/depression-induced mice, can be achieved through introducing *Lactobacillus mucosae NK41 *and *Bifidobacterium longum NK46*. The mixture of NK41 and NK46 also significantly reduced immobilization stress-induced anxiety-like/depressive behaviors [15]. Decreased expression of depressive-like behavior through the presence of *Lactobacillus rhamnosus* in chronically stressed mice was observed in work by Yong et al. [16]. These studies support the concept of the benefits of specific *Lactobacillus *in treating MDD through anti-inflammatory mechanisms or through encouraging serotonin production. Further exploration of the introduction of *Lactobacillus *through probiotics and its possible synergistic effects with SSRIs can strengthen the treatment regimen for MDD.

The role of the vagus nerve

The vagus nerve is the backbone of relaying communication between the gut and the brain. Hence, the vagus serves as the axis establishing the gut-brain axis phenomena [17]. Given the abundant pool of serotonin present in the stomach and the central role of the vagus nerve, how these factors respond to SSRIs highlights the presence of the gut-brain axis. McVey Neufled et al. explored the efficacy of SSRIs in both controls versus vagotomized mice. In the control, it was observed that SSRIs orally administered showed increased vagal activity apparent through the mesenteric nerve recordings. The antidepressive effects from the SSRI treatment were absent in the subdiaphragmatic vagotomized mice, determined by the tail suspension test [6]. Through these findings it is apparent that despite the use of SSRIs, without the vehicle of the vagus nerve the SSRIs have found to be ineffective. Bravo et al. also made evident that subdiaphragmatic vagotomy also stunted* L. rhamnosus*’s anxiolytic effects in mice. Bravo et al. was able to narrow the role of the vagus specifically into the gut-brain axis as the vagotomies in the mice were done subdiaphragmatically [18]. Seeing the vagus nerve's essential role in the gut-brain axis and how SSRIs and anxiolytic bacteria show dependence on the vagus nerve, the integrity of the vagus is crucial in providing effective management.

Gut-brain axis beyond depression

The phenomena of the gut-brain axis go beyond the realm of depression and can be seen active in irritable bowel syndrome (IBS), vertically during pregnancy, with psychiatric conditions and medications, autism, and many more disorders. IBS is regarded as the prototypic disorder in reference to the gut-brain axis due to its responsiveness to probiotics [19]. Studies have made it evident that IBS patients can benefit from SSRIs. Stasi et al.'s longitudinal examination of IBS patients taking fluoxetine, an SSRI, versus controls demonstrated that fluoxetine improved gastrointestinal and psychiatric symptoms in the study subjects [20]. The improvement of symptoms, both psychiatrically and gastrointestinal, exemplifies that mental screening in all IBS patients can provide better care. SSRIs are used in up to 10% of women for depression in pregnancy which can impact fetal brain development and the gut-brain axis [21,22]. Parallel to the impact SSRIs have on the gut-brain axis, Davey et al. finds with the antipsychotic, olanzapine, results suggest that the metabolic dysfunction induced by olanzapine can be ameliorated providing new targets for antipsychotic-induced metabolic disease [23]. Sherman et al. explore the benefits of understanding the gut-brain axis in neonates. Their work mentions improvements in preterm infants with necrotizing enterocolitis who have received care with probiotic bacteria. Probiotics have also been demonstrated by researchers to block the transport of damaging biomolecules and thus prevent brain injury [4]. Clarke et al. also discuss the role of the gut-brain axis in the immune and endocrine system maturation and find evidence to suggest that a restoration of a normal gut in later life may not be a restorative measure [24]. The importance of the role of the gut-brain axis is evident from preterm infancy to well beyond adulthood. Research shows that the manipulation of the gut-brain axis can be therapeutic in many diseases including and not inclusive of Parkinson’s, obesity, autism, and multiple sclerosis [25,26,27]. Santos et al. found that the hallmark finding of Parkinson’s disease, the aggregates of alpha-synuclein in the central and peripheral nervous system, can also be produced by gut toxins and via the vagus transmitted to the CNS [25]. The coexistence of gastrointestinal problems with autism spectrum disorders (ASD) and the role of the gut-brain axis have been explored by Srikantha et al. The results indicate a lack of diversity in the microbial content of ASD children as opposed to controls. Beyond the lack of diversity, there is a decrease in *Bacteroidetes *to *Firmicutes *ratio, along with other specificities to the microbiota of ASD. Srikantha et al. identified specific characteristics of the microbiota of ASD similarly to how the microbiota of MDD have been given a signature [26]. Once again the role of the gut microbiota is visible through the development of mood disorders and ASD [28]. Understanding the interplay of probiotics, microbiota, and the gut-brain axis can provide new avenues for prevention and treatment in many disease processes throughout various aspects of medicine that can benefit patients from infancy through adulthood.

## Conclusions

Understanding the utilization of gut microbiota concerning depression and antibiotics is a growing area of interest. Many studies show promising evidence of implications of how tailoring gut microbiota can optimize management for depression patients. Research shows that bacteria that are consistent with a depressive diagnosis can be narrowed to a specific stool signature. This defined phenotype allows researchers to focus on incorporating it into diagnostics, especially in regards to many psychiatric illnesses that currently have no quantifiable means. Potential treatments with fecal transplants are also introduced in the management of MDD where the side effect profile is at a minimum. Probiotics containing specific characteristic bacteria have proven to have favorable outcomes when co-administered with SSRIs. The gut-brain axis enables these findings to be observed. The importance of the existence of the gut-brain axis to allow the relay of these implications has been found through experimentation of vagotomized versus unvagotamized mice. SSRIs' benefits through the gut-brain-axis for IBS patients have shown promising results, indicating that working towards a healthy gut microbiota can provide better management. Many studies have shown that establishing a stool signature for depression can not only be part of the diagnostics that presently heavily rely on patient interviews but can narrow differentials. Identifying the depression stool signature can allow researchers to create and administer probiotics to reconstruct a healthy gut microbiota. Research is being done on the interplay of antidepressants and gut microbiota, expanding the understanding of the gut ecosystem to include and identify parallels between viruses and fungi that can further complete our comprehension. It would be of great interest to have further research to explore healthy diets, lactose-free diets, the role of viruses and fungi in the gut-brain axis and continue research on how stool signatures can be used as part of the diagnostics of depression serving as an evidence-based approach.
